# 

**Published:** 1856-06

**Authors:** 


We would respectfully, but earnestly, call the attention of our subscribers and others having business arrangements with the Journal to the present distribution of the labors and duties in connexion with its publication.

The editorial department is a very onerous one when taken in connection with other and pressing labors. But when the business matters are super-added, it is more than can be well attended to, and therefore, one or the other must be neglected. The propriety of the division of the labors has been apparent all along, but recently it has become imperative. An individual was chosen as a "business committee" to whom all such communications should be directed. Besides this another was added to the editorial committee, and yet this last department has too much labor to perform.

All persons therefore, are again notified that they should communicate with Prof. J. C. Hughes, M. D., on all business relations, and all communications for publication to be addressed to the editors.

Our subscribers will not fail to observe this for although one may conclude that their letter will not cost much trouble to answer it, yet when a number of such come to hand, all must be examined and all answered, or many of them, which is no small labor and consumes much time.

